# Heavy Metals and Polycyclic Aromatic Hydrocarbons in Soil from E-waste Dumpsites in Lagos and Ibadan, Nigeria

**DOI:** 10.5696/2156-9614-7.15.71

**Published:** 2017-09-07

**Authors:** Adebola Abosede Adeyi, Peter Oyeleke

**Affiliations:** Department of Chemistry, University of Ibadan, Ibadan, Nigeria

**Keywords:** e-waste, soil contamination, heavy metals, PAHs, ecological risk, waste management

## Abstract

**Background.:**

Soil contamination from heavy metals and polycyclic aromatic hydrocarbons (PAHs) released during informal e-waste processing and disposal poses human and ecological health risks in Nigeria.

**Objectives.:**

This study assesses the levels of heavy metals and PAHs in soils of e-waste dumpsites in Lagos and Ibadan, Nigeria.

**Methods.:**

Composite soil samples were collected at depths of 0–15 cm, 15–30 cm and 30–45 cm from major e-waste dumpsites in Lagos and Ibadan and analyzed for lead (Pb), cadmium (Cd), copper (Cu), nickel (Ni), zinc (Zn), chromium (Cr) and PAHs to evaluate the potential contaminant contribution from e-waste activities. Control samples were collected at the Botanical Garden, University of Ibadan. Samples were analyzed for heavy metals after acid digestion using atomic absorption spectrophotometry, while PAHs were extracted using cold solvent extraction and quantified by gas chromatography-mass spectrometry. Blank determination and recovery studies were carried out for each metal. Contamination and ecological risks were assessed using soil contamination indices such as contamination factor, geo-accumulation and pollution load indices, and potential ecological risk index to categorize contaminant concentrations and associated impacts. Soil physico-chemical characteristics such as pH and total organic matter were also determined.

**Results.:**

Metals concentrations in the dumpsite soils ranged from 114–2,840 mg/kg and not detectable - 6.50 mg/kg for Pb and Cd, and 42.8–5,390 mg/kg, 27.5–3,420 mg/kg, 11.0–128 mg/kg and 94.0–325 mg/kg for Cu, Zn, Ni and Cr, respectively. Serious metals accumulation was observed at every e-waste dumpsite, as shown by the pollution load index. The potential ecological risk values were between 584 and 10,402 at all of the dumpsites, signifying very high ecological risk. The total PAHs ranged from 1,756–2,224 μg/kg at the 0–15 cm level, 1,664–2,152 μg/kg at 15–30 cm and 278 μg/kg in the top- and sub-soil of the control site.

**Discussion.:**

The total PAHs in the soil of e-waste dumpsites was significantly higher than in the control soil.

**Conclusions.:**

The results of this study indicate that indiscriminate dumping and open burning of e-waste are potential sources of PAH and toxic metal emissions, which can pose serious human health and ecological risks.

## Introduction

Soil contamination resulting from uncontrolled dumping of municipal, industrial, and agricultural solid waste, as well as hazardous waste such as e-waste, has become a public health concern in Nigeria.^1–6^ Of particular concern is soil contamination at informal electronic waste recycling and disposal sites. In Nigeria, domestic and imported e-waste streams are growing steadily due to the increased availability of secondhand computers used in computer training centers, printing houses, cyber cafes, business centres and homes. Researchers have estimated that, on average, 500 shipping containers, with 400,000 computer monitors or 175,000 large TV sets enter the port of Lagos, Nigeria per year. As much as 75% of this waste is unserviceable and unable to be refurbished, and thus becomes e-waste.^7–10^

In addition to precious metals such as gold, silver, and platinum, e-waste contains toxic metals such as lead (Pb) and cadmium (Cd), arsenic (As), and mercury (Hg).^11^ Informal e-waste recycling and disposal practices such as open burning and dumping can lead to leaching of these toxic metals into the soil. Humans can be exposed to soil contaminants from e-waste dumpsites through accidental soil ingestion or direct dermal exposure.^12–21^ Lead levels in dust have been significantly associated with Pb levels in children's blood, and a blood lead level greater than 10 μg Pb/dL has been associated with a decrease in intelligence quotient (IQ).^22^,^23^ Exposure to high levels of heavy metals such as Pb, Cd, and Hg through ingestion and dermal contact can result in acute and chronic toxicity. These metals can damage the central and peripheral nervous systems, result in blood abnormalities, impair the lungs, kidneys, and liver, and even lead to death. The health and environmental effects of individual metals vary from toxic to endocrine disruption.^24–27^ Elevated metals concentrations in surface soils can pose a risk to human health.^28^ Heavy metals can migrate from surface soil to subsoil and contaminate ground water. They can also bio-accumulate in the food chain, posing health risks at high concentrations.

Polycyclic aromatic hydrocarbons, another class of toxic chemicals, are released by low-temperature combustion of e-waste.^19^,^29^,^30^ Although limited data exist on the distribution and transport of polycyclic aromatic hydrocarbons (PAHs) from e-waste dumpsites in Nigeria, PAHs are known to be lipophilic and accumulate in the food chain near contaminated sites.^20^,^31–37^ Their lipophilicity also makes dermal absorption possible. Epidemiological studies on occupational exposure to PAHs indicate that they can contribute to induction of skin and lung cancers. It has been reported that certain PAH metabolites interact with deoxyribonucleic acid (DNA) and are genotoxic, causing malignancies and heritable genetic damage in humans.^38^ The lower molecular weight PAHs (e.g., 2–3 rings) such as naphthalene, fluorene, phenanthrene and anthracene have significant acute toxicity to aquatic organisms, while higher molecular weight PAHs (4–7 rings) such as chrysene and coronene do not, but are carcinogenic.

This study assesses the distribution and levels of toxic metals and PAHs in the soil of selected e-waste dumpsites in Lagos and Ibadan, Nigeria, where open burning is prevalent. Data of this nature are currently lacking for Nigeria, and understanding local contaminant levels is important for effective health risk assessment. We also estimated human and ecological health risks using the pollution load index and ecological risk index, using our soil concentration data as inputs.^39^,^40^ A secondary objective was to determine contaminant origin (lithogenic versus anthropogenic) using the index of geo-accumulation and contamination factors.^39^,^41^

## Methods

### Study Area

Lagos and Ibadan are located in southwestern Nigeria. Alaba international market, Ojo (LLS_1_) and Chinatown, Ojota (LLS_2_) are the locations of the two e-waste dumpsites selected for the present study in Lagos. The Alaba market sampling site is a large expanse of land adjacent to the market shopping complex. The major wastes observed on this site were e-waste, followed by polythene bags, cartons, cardboards and cans. The Chinatown dumpsite is located on a small plot of land adjacent to the Chinese building at Ojota, a suburb in Lagos. Wastes observed there included broken monitor glass, plastics, cans, polythene bags and paper. The three Ibadan dumpsites include along Iwo road/Ile-pupa, located behind an electronics shopping complex (ISS_1_), the Ogunpa dumpsite, adjacent to the Ogunpa River channel (ISS_2_), and the Dugbe dumpsite, adjacent to residential buildings (ISS_3_). At every site except for Dugbe (ISS_3_), open burning to recover copper and other valuable materials is commonly practiced. At Dugbe, no traces of burning were apparent among the e-waste piles. Control samples were also collected at the Botanical Garden, University of Ibadan, Ibadan.

### Soil Sample Collection

Samples for PAH determination were collected with a stainless steel hand trowel, while plastic was used for collection of samples for heavy metal determination. The stainless hand trowel and plastic were cleaned thoroughly to prevent cross contamination. Samples were collected randomly at almost 5 m distance from five different points and combined to form a composite sample, with this process repeated at three different depths (0–15 cm, 15–30 cm and 30–45 cm) for heavy metal determination and two depths (0–15 cm and 15–30 cm) for PAH determination. Samples for PAHs were packed in pre-cleaned aluminum foil, which was previously solvent rinsed and dried at 80°C. Polyethylene bags were used for packing soils for heavy metal determination. Samples for metals and soil characteristics determination were air-dried in the laboratory after manual removal of stones, twigs and other large materials then ground in a porcelain mortar and passed through a 2-mm sieve. PAH samples were preserved on ice and kept in the refrigerator prior to extraction and analyses.

**Abbreviations**
*^C^_f_^i^*: Contamination factor
*Eir*: Ecological risk factors
*HMW*: High molecular weight
*I_geo_*: Geo-accumulation index
*LMW*: Low molecular weight
*PAH*: Polycyclic aromatic hydrocarbon
*PLI*: Pollution load index
*RI*: Ecological risk index
*VROM*: Dutch Ministry of Housing, Spatial Planning and the Environment

### Analytical Procedures

Samples were analyzed for PAHs, heavy metals and soil characteristics. For the metals analysis, approximately 1 g each of the sieved samples were weighed into digestion tubes and 10 ml aqua regia (concentrated hydrogen chloride and nitric acid, ratio 3:1 vol/vol) added (United States Environmental Protection Agency (USEPA) method 3050b).^42^ The tubes were covered, heated in a water bath to 100°C for 2 hours with intermittent shaking, cooled to room temperature, and then filtered using filter papers (pore size 110 mm). The filtrate was diluted with distilled water to 25 mL and analyzed for total Pb, chromium (Cr), Cd, nickel (Ni), zinc (Zn) and copper (Cu) using atomic absorption spectrophotometry (Buck Scientific Model 205A). Metal recovery was carried out by spiking 1 g of the soil sample with known concentrations of each metal. The concentrations of the metals were determined after taking the spiked sample through the entire procedure. The concentrations of each metal in the unspiked sample was deducted from that of the spiked sample and divided by the concentrations of the metals used for spiking, then multiplied by 100. The recovery was between 93.2 -100.4% for all the metals.

Sixteen target PAHs were analyzed using gas chromatography-mass spectrometry (GS/MS) following modified USEPA methods (method 827°C).^43^ Approximately 5 g of each sample and 5 g of anhydrous sodium sulphate were weighed and homogenized to a complete mixture. The mixtures were transferred to pre-cleaned extraction tubes, and 25 mL dichloromethane added. The tubes were tightly capped, allowed to stand for 30 minutes, and then shaken vigorously for 30 minutes. The solids were allowed to settle and solvent layers were filtered using filter papers. The procedure was repeated with 25 mL dichloromethane. The two extracts were combined, concentrated on a rotary evaporator (Büchi Rotavapor R-114), exchanged with 5 mL of n-hexane and re-concentrated to 1 mL for clean-up. The extracts were then eluted with 25 mL dichloromethane/hexane (20:80 v/v) on a silica gel column. The extracts were evaporated and re-dissolved in 1 mL n-hexane. The cleaned extracts were analyzed for the 16 representative PAHs using a Shimadzu GS/MS QP 2010 model. Helium gas was used as the carrier gas with a constant flow rate of 1 mL/min, HP-1 ms column (30 m × 0.25 μm 0.25 mm ID), injection mode was pulsed splitless, volume of extract injected was 1 μL, injection port temperature was 290°C, pulse pressure and flow were 35 psi (0.5 min) and 20 mL/min (2 min), respectively; solvent delay was 5 min, initial oven temperature and hold time was 50°C (1 min), ramped at 30°C/min to 280°C and 15°C/min to 310°C with final hold time of 4 min. External calibration using PAHs standard was used for analytes quantification, while identification was based on retention time. The quantification limit of the PAHs in the standard and the samples was 0.001 ppm. The average response factor for the weight ranges were calculated and used for sample quantification. The concentration of each analyte was determined by calculating the amount of analyte injected from the peak response in area ratio as shown below:

Calibration factor for each priority PAH =: A_C_/M_c_
Average calibration factor for each priority PAH, CFav =: (ΣCF)/N
The amount of analyte injected, Xs =: A_S_/CF_av_
Actual concentration of the analyte in the sample extracted (µg/kg) =: Xs x Vt x Df/Ws

Where;

A_c_ =: peak area of the compound in the standard
M_c_ =: mass of the compound injected in nanograms
N =: number of calibration points in the external calibration curve
A_S_ =: peak area of the analyte in the sample
CF_av_ =: average calibration factor (for each analyte, the average of the different calibration points)
X_s_ =: calculated mass of the analyte in the sample aliquot introduced into the instrument (in nanograms)
V_t_ =: total volume of the concentrated extract (μL)
W_s_ =: weight of soil sample extracted (g)

Soil pH and total organic carbon (TOC) were determined by standard methods using a Jenway 3310 pH meter in ratio 1:2 (wt/vol) and the Walkey-Black method, respectively.^44^ Approximately 0.5 g of each of the sieved samples were weighed, 10 mL of standard potassium dichromate solution added, and swirled to mix, 15 mL of concentrated sulphuric acid was added gently and mixed. The flasks were allowed to stand for 30 minutes. Five drops of ferroin indicator was added and the resulting mixtures were titrated against ferrous ammonium sulphate until color change from blue green to violet red was observed. Total organic carbon was determined using an appropriate mathematical expression and multiplied by a factor to obtain the total organic matter (TOM).^22^,^24^

### Soil Contamination

The degree of contamination of the dumpsite and the control site soils was evaluated using four indices.

#### Geo-accumulation Index

Geo-accumulation Index (I_geo_) shows the degree of anthropogenic pollution in soil samples by comparing soil metals concentrations to average shale values.^41^,^45^ It is expressed using Equation 1

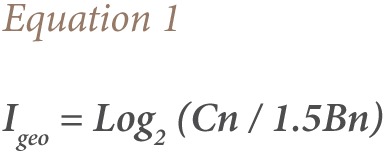
where,

Cn is the measured concentration of a particular metal in a particular soil sample; Bn is the geochemical background value in average shale of element n and 1.5 is a background matrix correction factor, accounting for lithogenic effects.^46^ We then classified each I_geo_ using Forstner et al. descriptive categories: <0, unpolluted; 0–1, unpolluted to moderately polluted; 1–2, moderately polluted; 2–3, moderately to highly polluted; 3–4, highly polluted; 4–5, highly to very highly polluted, and >5, very highly polluted.^47^

#### Contamination Factor

The contamination factor (C_f_^i^) was used by Hakanson to assess soil contamination by comparing the contaminant concentration in the surface layer to a background value.^39^,^48^ We used a modified C_f_^i^ formula, using metals concentrations in the control samples instead of background values, which are currently lacking for Nigeria.^49^ It is expressed using Equation 2.

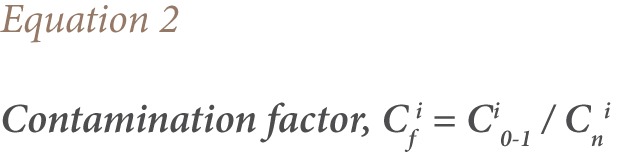
where,

C_f_^i^ = contamination factor; C^i^_0–1_ = mean concentration of each metal in the soil; Cn^i^ = baseline or background value (concentration of each metal in the control sample was used); n = number of analyzed elements; i = ith element (or pollutants). We then classified the C_f_^i^ using descriptive categories: C_f_^i^ < 1, low contamination; 1 ≤ C_f_^i^ < 3, moderate contamination; 3 ≤ C_f_^i^ < 6, considerable contamination; and 6 ≤ C_f_^i^, very high contamination.

#### Pollution Load Index

Pollution load index (PLI) was also used to assess the metal accumulation and multi-element contamination resulting in increased overall metal toxicity.^50^ Heavy metal contamination is associated with a mixture of contaminants rather than one metal contaminant.^51^ The higher the pollution load index, the more serious the heavy metal accumulation in the soil.^50^ We used the PLI to characterize the aggregate contamination of the six target metals using Equation 3.

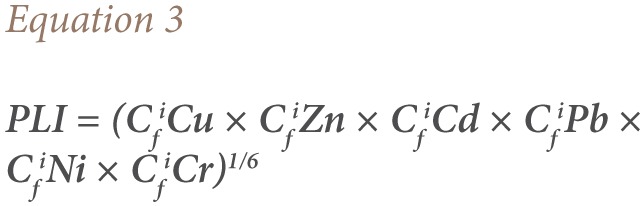
Where; C_f_^i^ is the contamination factor of each metal.^40^

#### Potential Ecological Risk Index

In this study, a simplified approach to risk assessment based on comparison of the measured level of contamination in the soil of the studied sites with the background value from the control sample was adopted.^49^ Although the ecological risk index (RI) is primarily intended by Hakanson to express the ecotoxic potential of increased concentrations of toxic metals such as arsenic, Cu, Ni, cobalt, Pb, Cd, and mercury in consumable fish, it can also be applied for the assessment of the potential risk from toxic substances to biota and non-human biota in other similar media such as contaminated soils.^39^,^52^ We used the RI introduced by Hakanson to characterize the metal contamination of each sample in terms of their potential ecotoxicity using the Equations 4 to 6.

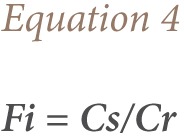


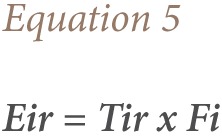


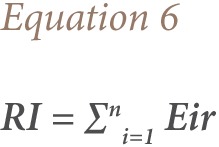
where;

Fi is the single metal pollution index; Cs is the concentration of metal in the samples; Cr is the reference value for the metal; Eir is the monomial potential ecological risk factor; Tir is the metal toxic response factor according to Hakanson, and Zn = 1< Cr = 2 < Cu = Ni = Pb = 5 < As = 10 < Cd 30.^39^,^53^ The ecological risk index is the potential ecological risk caused by the overall contamination categorized in the four classes as shown in Table 1. The potential ecological risk caused by Cu, Zn, Cd, Pb, Ni and Cr on the e-waste dumpsite soils in Lagos and Ibadan were calculated based on the potential ecological risk factor (Eir). The ecological RI value characterizes the sensitivity of the local ecosystem to the pollutants i.e., metals, and represents the ecological risks resulting from the overall contamination. The overall RI was calculated as the sum of all the four risk factors.

**Table 1:** Ecological Risk Category of Metals

Eir value	Level of Ecological Risk of Metal	RI Value	Ecological Risk Category
Eir < 40	Low risk	RI < 110	Low risk
40 ≤ Eir < 80	Moderate risk	110 ≤ RI < 200	Moderate risk
80 ≤ Eir < 160	Considerable risk	200 ≤RI <400	Considerable risk
160 ≤ Eir < 320	High risk	400 ≤ RI	Very high risk
320 ≤ Eir	Very high risk		

Abbreviations: RI, ecological risk index; Eir, ecological risk factor

### Statistical Analysis

Obtained data (i.e., soil properties, metals concentrations and total concentrations of PAHs) were subjected to descriptive statistics and Pearson's correlation coefficient to determine whether there were significant relationships between total PAHs, metals concentrations and soil properties. The statistical analysis was performed using Statistical Package for Social Sciences (SPSS) version 16.0.

## Results

### Soil Characteristics and Total Metals Concentrations

The pH of topsoil (0–15 cm) ranged from 5.77–5.80 and 5.84 - 6.30, respectively, in samples collected in Lagos (LSS) and Ibadan (ISS), while total organic matter ranged from 8.32–8.85% and 3.27–8.65%, respectively (*Table 2*). In general, e-waste dumpsite soils were more acidic than the control soil. This might be attributed to the parent material and burning of wastes on the dumpsites. Among dumpsite soils, the Lagos samples were more acidic, with high TOM compared to Ibadan samples. Metals concentrations across dumpsites varied widely. Topsoil Pb ranged from 193–2,240 mg/kg in Lagos and 246–2,090 mg/kg in Ibadan, while Cu ranged from 50.5–5,390 mg/kg and 79.3–1,150 mg/kg, Zn ranged from 220–1930 mg/kg and 27.5–3420 mg/kg, Cd ranged from 0.43–5.85 mg/kg and not detectable-6.50 mg/kg, Ni ranged from 11.0–51.5 mg/kg and 27.7–128 mg/kg, and Cr ranged from 108–118 mg/kg and 94.0–325 mg/kg, in Lagos and Ibadan, respectively (*Table 2*). Metals concentrations in the control sample were generally lower than what was detected in e-waste dumpsite soils by over one hundred orders of magnitude in some metals, which may be at least partly explained by e-waste burning activity at the dumpsites. Most heavy metals determined in soils collected from Wenling, an emerging e-waste recycling city in Taizhou, China exceeded the respective Grade II value of soil quality standards from the State Environmental Protection Administration of China and also exceeded the Dutch optimum values.^19^ High levels of Cu (712 and 496 mg/kg) exceeding the new Dutch list action value (of 190 mg/kg) were reported in soil near a printer roller dumping area and a plastic burning site at an electronic waste recycling site at Guiyu, southeast China (*Table 3*).

**Table 2:** Concentrations of Heavy Metals and Soil Characteristics of E-waste Dumpsites in Lagos and Ibadan

**Sampling Location**	**Depth (cm)**	**Cu**	**Zn**	**Cd**	**Pb**	**Ni**	**Cr**	**pH**	**TOM (%)**
**Lagos**									
LLS_1_	15	5,390	1,820	5.63	2,840	51.5	113	5.77	8.85
	30	3,830	1,550	5.85	1,630	36.1	110	5.77	8.63
	45	2,780	1,480	2.63	260	25.1	108	5.76	6.73
LLS_2_	15	50.5	220	0.45	193	17.5	114	5.80	8.32
	30	57.5	1,930	0.58	114	12.0	118	5.77	6.88
	45	50.0	1,750	0.43	142	11.0	111	5.77	3.48
**Ibadan**									
ISS_1_	15	1,150	3,420	4.53	2,090	48.7	114	5.93	8.65
	30	440	161	2.28	768	27.7	107	5.88	6.79
	45	310	1,430	1.98	302	40.5	104	5.89	3.09
ISS_2_	15	101	1,840	6.30	1,030	46.4	103	5.84	7.95
	30	356	571	2.88	365	27.7	111	5.88	6.54
	45	1,210	815	6.50	840	41.1	105	5.89	3.06
ISS_3_	15	79.3	105	0.03	246	128	325	6.30	3.27
	30	51.8	77.8	0.03	253	104	117	6.17	0.88
	45	42.8	27.5	ND	178	47.5	94.0	6.10	0.36
CSS	15	3.98	47.4	0.35	6.25	0.33	3.95	6.89	1.56
	30	7.55	26.2	0.45	6.75	2.35	3.58	7.21	0.29
	45	4.75	19.8	0.50	13.8	2.23	5.08	7.07	0.24

Values presented as mg/kg

Abbreviations: ND, not detected

**Table 3:** Comparison of Heavy Metal Concentrations in Soil of E-waste Dumpsites Across Studies

Location	Type of Soil or Sediment		Cr	Ni	Cu	Zn	Cd	Hg	Pb	Reference
			mgkg^−1^ dry weight							
**Lagos, and Ibadan**, **Nigeria**	E-waste dumpsite soils									This study
	Surface soil (0–15 cm)	Range	103–325	17.5–128	50.0–5,390	105–3,420	0.03–6.30	-	246–2,840	
	Subsoil (15–30 cm)	Range	107–118	12.0–104	51.8–3,830	77.8–1,930	0.03–5.85	-	114–1,630	
	Subsoil (30–45 cm)	Range	47.5–111	11.0–47.5	42.8–2,780	27.5–1,750	ND-6.50	-	142–840	
Taizhou, Zhejiang Province, China	Paddy soil (0–20 cm) in an e-waste recycling area (*n* = 6)	Range	54.4–74.1	25.8–46.2	56.1–236.9	-	0.55–7.86	0.24–0.76 5	1.96–64.6	53
Longtang Town, Northern Guangdong Province, China	Surface soil (0–15 cm) of a vegetable garden (n = 16)	Range	12.3±5.1	8.83±2.9	324±172	122±55.7	0.9±0.8	-	95.6±19.5	54
			9.66–19	7.04–10.3	210–450	92.4–142	0.26–1.17	-	73.3–134	
	Surface soil (0–15 cm) of a paddy field (n = 11)	Range	17.3±8.1	34.5±26.6	155±94	166±76.7	1.0±0.4	-	61.8±24	
			10.5–24.1	10.8–66	40.1–260	62.1–252	0.04–1.43	-	48.1–97	
	Surface soil (0–15 cm) of an incineration site (n = 11)	Range	68.9±53	60.1±59	11,140±9,000	3,690±2,680	17.1±12.5	-	4,500±3 370	
			23.6–122	12.2–132	1,500–21,400	682–8,970	3.05–46.8	-	629–7,720	
Bangalore City, India	Soil of an e-waste recycling site in a slum area (n = 7)	Range	46–160	-	61.7–4,790	126–2,530	0.385–38.9	0.09–59	90.4–2,850	55
	Soil of an e-waste recycling area (n = 3)	Range	50–62	-	154–2,190	119–499	0.301–0.906	-	79.1–262	

### Metals Contamination Indices

The I_geo_ analysis showed that the soil of LLS_1_ was very highly polluted with Pb and Cu, moderately to highly polluted with Zn and Cd, and unpolluted with Cr and Ni (*Table 4*). The second dumpsite in Lagos, LLS_2_, was highly polluted with Zn and Pb, unpolluted with Cu, Ni, and Cr, and unpolluted to moderately polluted with Cd. The same trend was observed in Ibadan dumpsites samples, while the control sample was not found to be polluted with any of the target metals. The Dugbe dumpsite (ISS_3_) in Ibadan, where e-waste burning was not typically observed, was generally unpolluted with the targeted metals except for Pb (_Igeo_ range 2-3) and Ni (I_geo_ range 0–1), which may be attributed to other possible sources of contamination such as vehicular emissions and atmospheric deposition.

**Table 4:** Geo-accumulation Indices of Metals in Dumpsite and Control Soil Samples

**Sampling Location**	**Depth (cm)**	**Cu**	**Zn**	**Cd**	**Pb**	**Ni**	**Cr**
**Lagos**							
LLS_1_	15	6.32	3.67	3.65	6.56	0.99	−0.26
	30	5.83	3.44	3.70	5.76	−1.50	−0.30
	45	5.36	3.38	2.55	3.11	−2.02	−0.32
LLS_2_	15	−0.42	0.63	3.2 × 10^−16^	2.69	−2.54	−0.24
	30	−0.23	3.76	0.37	1.93	−3.09	−0.19
	45	−0.43	3.62	−0.07	2.24	−3.21	−0.28
**Ibadan**							
ISS_1_	15	3.09	4.58	3.33	6.12	−1.07	−1.47
	30	1.70	0.18	2.34	4.68	−1.88	−2.29
	45	1.20	3.33	2.14	3.33	−1.33	−1.74
ISS_2_	15	−0.42	3.69	3.81	5.10	−1.14	−1.54
	30	1.40	2.00	2.68	3.60	−1.88	−2.29
	45	3.16	2.52	3.85	4.81	−1.31	−1.72
ISS_3_	15	−0.77	−0.44	−4.17	3.04	0.38	−0.08
	30	−1.38	−0.87	−4.17	3.08	0.03	−0.38
	45	−1.66	−2.37	0	2.57	−1.10	−1.51
CSS	15	−4.08	−1.59	−0.36	−2.26	−8.27	−5.09
	30	−3.16	−2.44	3.2 × 10^−16^	−2.15	−5.44	−5.24
	45	−3.83	−2.85	0.15	−1.12	−5.52	−4.73

The C_f_^i^ analyses using Hakanson's classification showed that the dumpsites soils were highly contaminated with all of the metals except for Cd, which showed moderate contamination at the sites LLS_2_ and ISS_3_ (*Table 5*).^39^ Contamination of site LLS_1_ was the highest, possibly attributable to the large volume of e-waste handled at Alaba, an international electronics market for both Nigeria and West Africa. The PLI calculations showed serious metals accumulation at all the e-waste dumpsites with the Alaba market (LLS_1_) having the highest pollution load index of 109 (*Table 5*). In the Eir analyses, Zn showed low levels of ecological risk at all the dumpsites except for ISS_1_ in Ibadan, which had moderate risk (*Table 6*). In most cases, Cu, Cd, Pb, and Ni showed moderate to very high ecological risk, while Cr showed moderate to high ecological risk. The RI values were between 584 and 10,402 at all of the dumpsites, signifying very high ecological risk.

**Table 5:** Metal Contamination Factors and Pollution Load Indices in Dumpsites

**Sites**	Cu	Zn	Cd	Pb	Ni	Cr	Total C^f^_i_	PLI
**Lagos**								
LLS_1_	1354	38.4	16.1	454	156	28.6	2,047	109
LLS_2_	12.7	4.64	1.29	30.9	53.0	28.9	131	12.4
**Ibadan**								
ISS_1_	289	72.2	12.9	334	148	28.9	885	85.1
ISS_2_	25.4	38.8	18.0	165	141	26.1	414	46.9
ISS_3_	19.9	2.22	0.07	39.4	388	82.3	532	12.6

Abbreviations: Cf

i, contamination factor; PLI, pollution load index

**Table 6:** Metals Ecological Risk Factors and Risk Indices in Dumpsites

**E-waste Dumpsites**	**Eir**						**RI**

	**Cu**	**Zn**	**Cd**	**Pb**	**Ni**	**Cr**	
**LLS1**	6,771	38.4	483	2,270	780	57.2	10,402
**LLS2**	63.4	4.64	38.6	154	265	57.7	584
**ISS1**	1,445	72.2	388	1,672	738	57.7	4,373
**ISS2**	127	38.8	540	824	703	52.2	2,285
**ISS3**	99.6	2.22	2.14	197	1,939	165	2,405

Abbreviations: Eir, ecological risk factor; RI, risk index

### PAH Concentrations

The USEPA identified 16 priority PAHs, which can be classified as being of low or high molecular weight. Low molecular weight (LMW) PAHs (i.e., acenaphthylene, naphthalene, acenaphthene, fluorene, phenanthrene and anthracene), also referred to as petrogenic (formed during the emission of non-combustion-derived matter, including inadvertent oil spills), have molecular weights ranging from 128.2 to 178.2 g/mol. High molecular weight (HMW), pyrolytic PAHs, are fluoranthene, pyrene, benzo(a)anthracene, chrysene, benzo(b)fluoranthene, benzo(a) fluoranthene, benzo(k)pyrene, dibenzo(a, h)anthracene, benzo(g, h, i) perylene and indeno(1,2,3-cd) pyrene and have molecular weights ranging from 202.3 to 278.4 g/mol.^56^,^57^ Pyrogenic PAHs are formed during the incomplete combustion of coal, oil, gas wood and garbage. The % HMW PAHs was higher than % LMW PAHs at the dumpsites and the control site. The ranges of % LMW and HMW at 0–15 cm were 27.6–34.9 and 48.5–52.9, respectively, and 24.1–32.8 and 47.4–54.8, respectively, at the 15–30 cm depth. The percentage at both depths in the control soil was 18% and 65.5%, respectively (*Tables 7 and 8*). This indicates that high molecular weight PAHs were the predominant PAHs throughout the dumpsites. The total concentrations of the 16 target PAHs, including 5 carcinogenic PAHs, in the dumpsites and control soils at the 0–15 cm and 15–30 cm depths are presented in Tables 7 and 8, respectively. Total PAHs ranged from 1,756–2,224 μg/kg and 1,664–2,152 μg/kg at depths of 0–15 cm and 15–30 cm, respectively. Total PAHs obtained from the control soil was 278 μg/kg at both depths. Total PAHs were generally an order of magnitude greater in the dumpsites soils compared to the control sample, possible evidence of contamination by burning activities at the dumpsites. Anthracene and 2-methyl naphthalene were not detected in any of the samples, while phenanthrene, pyrene and fluoranthene were found in the highest concentrations for the PAHs determined in this study. Five and six-membered ring PAHs were the most prevalent at all of the sites, which may be due to their very high level of resistance to environmental degradation.^54^ Among the 7 carcinogenic PAHs (i.e., banz(a) anthracene, chrysene, benzo(a) fluoranthene, benzo(k)fluoranthene, benzo(a)pyrene, indeno(1,2,3-cd) pyrene and benzo(g,h,i)perylene), benzo(g,h,i)perylene was not detected in any of the soil samples except for soil collected at the ISS_2_ dumpsite at a 15–30 cm depth. Benzo(a) fluoranthene and benzo(a)pyrene were not determined in any of the samples. The % carcinogenic PAHs in the soils of e-waste dumpsites in Lagos and Ibadan ranged from 29.5–39.6 and 31.2–47.5 at the 0–15 cm and 15–30 cm level, respectively.

**Table 7:** Polycyclic Aromatic Hydrocarbon Concentrations in Dumpsite and Control Soil Samples, 0–15 cm Depth

Target Analytes	LLS_1_	LLS_2_	ISS_1_	ISS_2_	CSS
Naphthalene (Nap)	10.0	ND	ND	ND	ND
2-methyl Naphthalene (mNap)	ND	ND	ND	ND	ND
Acenaphthylene (Acy)	37.0	38.0	38.0	38.0	ND
Acenaphthene (Ace)	9.00	9.00	9.00	9.00	ND
Fluorene (Flu)	140	137	141	143	ND
Phenanthrene (Phe)	323	593	345	392	50.0
Anthracene (Ant)	ND	ND	ND	ND	ND
Fluoranthene (Fla)	251	293	240	260	41.0
Pyrene (Pyr)	255	384	245	271	37.0
Benz(a)anthracene (BaA)	227	247	235	227	45.0
Chrysene (Chr)	213	199	190	267	33.0
Benzo(k)fluoranthene (BkF)	215	211	204	224	40.0
Benzo(b)fluoranthene (BbF)	109	113	109	111	ND
Perylene (Per)	ND	ND	ND	51.0	6.00
Benzo(g,h,i)perylene (BghiP)	ND	ND	ND	ND	ND
Dibenz(a,h)anthracene (DahA)	ND	ND	ND	ND	26.0
Indeno(1,2,3-cd)pyrene (IcdP)	ND	ND	ND	119	ND

Total PAH	1,789	2,224	1,756	2,112	278
%LMW PAH	29.0	34.9	30.4	27.6	18.0
%HMW PAH	52.9	50.5	51.8	48.5	65.5
% C PAH	36.6	29.5	35.8	39.6	51.8

Values presented as μg/kg.

Abbreviations: LMW, low molecular weight; HMW, high molecular weight; C, carcinogenic PAH; ND, not detected

**Table 8:** Polycyclic Aromatic Hydrocarbon Concentrations in Dumpsite and Control Soil Samples, 15–30 cm Depth

Target Analytes	LLS_1_	LLS_2_	ISS_1_	ISS_2_	CSS
Naphthalene (Nap)	ND	ND	ND	ND	ND
2-methylNaphthalene (mNap)	ND	ND	ND	ND	ND
Acenaphthylene (Acy)	37.0	38.0	38.0	37.0	ND
Acenaphthene (Ace)	9.00	9.00	9.00	8.00	ND
Fluorene (Flu)	141	136	ND	140	ND
Phenanthrene (Phe)	294	523	392	324	50.0
Anthracene (Ant)	ND	ND	ND	ND	ND
Fluoranthene (Fla)	229	277	241	259	41.0
Pyrene (Pyr)	226	349	244	274	37.0
Benz(a)anthracene (BaA)	229	245	227	242	45.0
Chrysene (Chr)	188	214	204	227	33.0
Benzo(k)fluoranthene (BkF)	202	213	206	225	40.0
Benzo(b)fluoranthene (BbF)	109	113	110	110	ND
Perylene (Per)	ND	35.0	ND	59.0	6.00
Benzo(g,h,i)perylene (BghiP)	ND	ND	ND	195	ND
Dibenz(a,h)anthracene (DahA)	ND	ND	ND	ND	26.0
Indeno(1,2,3-cd)pyrene (IcdP)	ND	ND	ND	116	ND

Total PAH	1,664	2,152	1,671	2,116	278
% LMW PAH	28.9	32.8	26.3	24.1	18.0
% HMW PAH	52.4	50.4	54.8	47.4	65.5
% C PAH	37.2	31.2	38.1	47.5	51.8

Values presented as μg/kg

Abbreviations: LMW, low molecular weight; HMW, high molecular weight; C, carcinogenic PAH

According to the Dutch Ministry of Housing, Spatial Planning and the Environment (VROM), the total concentrations of ten VROM PAHs (napthalene, anthracene, phenanthrene, fluoranthene, benzo(a) anthracene, chrysene, benzo(a) pyrene, benzo(g,h,i)perylene, benzo(k) fluoranthene and indeno(1,2,3-cd) pyrene) in soil should not exceed the maximum value of 1000 μg/kg.^58^ The concentrations of nine VROM PAHs determined in the soil samples at depths of 0–15 cm and 15–30 cm exceeded this value. The concentrations of nine VROM PAHs (benzo(a)pyrene was not determined) ranged from 1,231- 1,543 μg/kg and 1,142–1,588 μg/kg at the depths of 0–15 cm and 15–30 cm, respectively. The Institute of Soil Science and Plant Cultivation (Pulawy, Poland) classification showed that soils with total PAH < 1,000 μg/kg dry weight (dw) can be considered to be unpolluted.^59^ The total PAHs concentrations of all of the samples in the dumpsite soils and the control exceeded the typical concentration of arable topsoil (around 200 μg/kg) in Sweden.^60^ The target established by the Dutch government for PAHs in uncontaminated soil is 20–50 μg/kg (dw).^61^ The total PAHs concentrations at depths of 0–15 cm and 15–30 cm in all the e-waste dumpsites in Lagos and Ibadan exceeded the 50 μg/kg limit. Thus, all of the study sites were considered to be highly polluted by PAHs.

The ratio of PAH profiles maybe used to track their origin as petrogenic, biogenic and pyrogenic sources.^20^,^62^,^63^ Petrogenic sources are characterized with the predominance of LMW PAHs (naphthalene, 2-methyl naphthalene, acenaphthylene, acenaphthene, fluorene, phenanthrene and anthracene) over the HMW PAHs (fluoranthene, pyrene, benz(a) anthracene, chrysene, benzo(a) pyrene, dibenz(a,h)anthracene). A ratio of LMW to HMW greater than 1 indicates a petrogenic source.^64^ In the soils of e-waste dumpsites in Lagos and Ibadan, we obtained a LMW/HMW range of 0.55–0.69 and 0.48–0.65 at the 0–15 cm and 15–30 cm level, respectively, which indicated pollution of pyrolytic origin (*Table 9*). The ratios of fluoranthene to fluoranthene plus pyrene, benzo(a) anthracene to benzo(a)anthracene plus chrysene and indeno(1,2,3-cd) pyrene to indeno(1,2,3-cd)pyrene plus benzo(g,h,i)perylene were also used for source identification.^19^,^30^,^65^,^66^ The ranges obtained were 0.43–0.50, 0.44–0.50; 0.46–0.55, 0.52–0.55; 0–1.0, 0–0.37, respectively, at the 0–15 cm and 15–30 cm levels, respectively, for these PAHs. These values indicated that PAHs had both pyrolytic and petrolytic origins. The results obtained in this study were compared with those in the literature and are presented in Table 10. It was reported that total PAHs in soil collected from Wenling, an emerging e-waste recycling area in Taizhou, China ranged from 371.8 to 1231.2 μg/kg, and relatively higher PAHs concentrations were found in soils taken from simple household workshops.^19^

**Table 9:** Diagnostic Ratio of Polycyclic Aromatic Hydrocarbons

**Soil depth**	**LMW/HMW**	**Fla/(Fla + Pyr)**	**BaA/(BaA + Chr)**	**IcdP/(IcdP + BghiP)**
**0–15 cm**				
LLS_1_	0.55	0.50	0.52	0
LLS_2_	0.69	0.43	0.55	0
ISS_1_	0.59	0.49	0.55	0
ISS_2_	0.57	0.49	0.46	1.0
**15–30 cm**				
LLS_1_	0.55	0.50	0.55	0
LLS_2_	0.65	0.44	0.53	0
ISS_1_	0.48	0.50	0.53	0
ISS_2_	0.51	0.49	0.52	0.37
**Control**	0.27	0.53	0.58	0

Abbreviations: Fla, fluoranthene; Pyr, pyrene; BaA, benz(a)anthracene; Chr, chrysene; IcdP, indeno(1,2,3-cd)pyrene; BghiP, benzo(g,h,i)perylen

**Table 10:** Concentrations of the 16 USEPA Identified Polycyclic Aromatic Hydrocarbons in Soil and Sediment Samples from E-waste Processing Areas in China Compared with this Study

Location	Type of Soil or Sediment	Range	Mean	Reference
		μg/kg dry weight		

**Lagos and Ibadan, Nigeria**	Topsoil (0–15cm) of e-waste dumpsite (*n = 20*)	1,756–2,224		This study
	Subsoil (15–30 cm) of e-waste dumpsite (*n = 20*)	1,664–2,152		
	Control (0–15 cm; 15–30 cm) (n = 5)	278		
Guiyu, Guangdong Province,	Surface soil (0–10 cm) of a burnt plastic dump site (*n* = 3)		428	34
	Surface soil (0–10 cm) near an open burning site (*n* = 8)		851	30
	Surface soil (0–10 cm) of an open burning site (*n* = 5)		2,065	
	Surface soil (0–10 cm) of an open burning site (*n* = 5)		1,066	67
	Surface soil (0–10 cm) of an open burning site (*n* = 5)		899.9	
	Surface soil (0–10 cm) of an open burning site (*n* = 5)		2,777	
Taizhou, Zhejiang Province, China	Surface soil (0–20 cm) of large recycling plants (*n* = 5)	128.8–6,687.2		68
	Surface soils (0–20 cm) of small recycling workshops (*n* = 3)	135.3–228.8		
	Surface soils (0–20 cm) of control sites (*n* = 3)	5.2–29.4		
	Local agricultural surface soil (0–20 cm) from an e-waste recycling facility (*n* = 10)	330–20,000		69
	Topsoil (0–30 cm) of large-scale e-waste recycling plants in Wenling (*n* = 14)	488.0–764.0		19
	Topsoil (0–30 cm) of large-scale gold recycling plants in Wenling (*n* = 5)	371.8–850.7		
	Topsoil (0–30 cm) of household e-waste recycling workshops in Wenling (*n* = 18)	730.5–1,231.2		
	Reference site (*n* = 1)		0.4	
	Near household e-waste recycling workshops	809–7,880		70
	Near industrial parks	2,820–3,020		
Qingyuan, Guangdong Province, China	Road soils mixed with deposited dust near dismantling workshops (*n* = 29)	190.8–9,156.0	2,689.1	71

### Statistical Analysis

Statistical analyses of the results obtained in the e-waste dumpsites in Lagos and Ibadan using Pearson's correlation coefficient (*Tables 11 and 12*) showed very strong and negatively significant correlations between total PAHs versus Cd (r = −0.955, p< 0.05), Ni (r = −0.973, p< 0.05) and TOC (r = −0.899, p< 0.05) in Ibadan, suggesting that these contaminants might have originated from similar sources, such as burning of e-waste at dumpsites. There was no significant correlation between total PAHs and TOC (r = −0.395, p< 0.05), and no significant correlation with most of the metals except for Zn (r = 0.648, p< 0.05) in soils of e-waste dumpsites in Lagos, suggesting different emission sources.

## Discussion

Migration of Cd from topsoil to the subsurface soil was observed in both the Lagos and Ibadan dumpsites. In most cases, Cu, Zn and Pb concentrations were highest in topsoil, which was evidence of recent/anthropogenic contamination, but with limited evidence of migration to the subsoil.^72^ This indicates that there is little risk of groundwater contamination at these sites. All of the e-waste dumpsites in Lagos and Ibadan exhibited multi-element contamination from anthropogenic inputs, most likely from e-waste burning activity. The indices of potential ecological risk were found in the following order at the different sites:

LLS_1_: Cu > Pb > Ni > Cd > Cr > Zn;
LLS_2_: Ni > Pb > Cu > Cr > Cd > Zn;
ISS_1_: Pb > Cu > Ni > Cd > Zn > Cr;
ISS_2_: Pb > Ni > Cd > Cu > Cr > Zn;
ISS_3_: Ni > Pb > Cr > Cu > Zn > Cd.

The total PAH concentrations of all of the samples in the dumpsite soils and the control exceeded the typical concentration of arable topsoil (around 200 μg/kg) in Sweden and the target established by the Dutch government for PAHs in uncontaminated soil of 20–50 μg/kg (dw).^60^,^61^ This is a public health concern, since several of the measured PAHs are considered probable human carcinogens (benz(a)anthracene, benzo(a)pyrene, benzo(k)fluoranthene, chrysene, dibenz(a,h)anthracene and indeno(1,2,3-c,d)pyrene) or possibly (benzo(a)fluoranthene, benzo(k) fluoranthene and indeno(1,2,3-c,d) pyrene).^73^ Indeno(1,2,3-c,d)pyrene and dibenz(a,h)anthracene were not detected in any of the samples except at a site in Ibadan, ISS_2_, and the control soil, respectively, at depths of 0–15 cm and 15–30 cm. Phenantherene (LMW) PAH is a thermodynamically stable compound mainly derived from petrogenic sources (from the release of uncombusted petroleum products such as gasoline, diesel fuel and fuel oil from vehicle traffic).^30^ Its predominance in the soil of e-waste dumpsites in Lagos and Ibadan and the control indicates a petrogenic source. Comparison of pollution in the four dumpsites considered in Lagos and Ibadan showed the following trend:

0–15 cm: LLS_2_ > ISS_2_ > LLS_1_ > ISS_1_
15–30 cm: LLS_2_ > ISS_2_ > ISS_1_ > LLS_1_

The PAHs profile pattern in soils of four e-waste dumpsites in Lagos and Ibadan were similar. In most cases, the concentrations of individual PAHs were higher in soil at the 0–15 cm level compared to soil at the 15–30 cm level. Phenanthrene and pyrene were the most abundant pollutants at all of the sites at the 0–15 cm and 15–30 cm levels, except at LLS_1_, where phenanthrene and fluoranthene were the most abundant, while 2-methylnaphthalene and anthracene were not detected in any of the e-waste dumpsites or the control site. However, pyrene, fluoranthene, benz(a)anthracene, and chrysene (HMW) typically have a pyrogenic source (from combustion of fossil fuels). Hence, the PAH profile in the soil of e-waste dumpsites in Lagos and Ibadan suggests both petrogenic and pyrogenic sources.

## Conclusions

The degree of contamination and ecological risk posed by metals in e-waste dumpsite soils in Lagos and Ibadan, Nigeria were evaluated in the present study. The results provide evidence that open burning, stockpiling, and other improper e-waste management practices may have resulted in toxic metal accumulation in soils of e-waste dumpsites in Lagos and Ibadan, corroborating previous results at e-waste dumpsites in other countries. Various metals contamination indices showed moderate to very high levels of contamination in the dumpsite soils, indicating potential threats to human and ecological health. We found PAHs at levels exceeding 1,000 μg/kg in dumpsite soils, suggesting anthropogenic contamination from both petrogenic and pyrogenic sources. It was previously shown that leachates from municipal solid waste dumpsites in Nigeria contain high concentrations of metals, PAHs and PCBs.^56^

Our work shows that improper e-waste handling at these sites may contribute additional metals and PAH contamination and highlights the need for regular soil monitoring at major dumpsites in Nigeria.
